# EEG complexity measures for detecting mind wandering during video-based learning

**DOI:** 10.1038/s41598-024-58889-9

**Published:** 2024-04-08

**Authors:** Shaohua Tang, Zheng Li

**Affiliations:** 1 School of Systems Science, Beijing Normal University, Beijing, China; 2 International Academic Center of Complex Systems, Beijing Normal University, Zhuhai, China; 3 Center for Cognition and Neuroergonomics, State Key Laboratory of Cognitive Neuroscience and Learning, Beijing Normal University, Zhuhai, China

**Keywords:** Neural decoding, Attention

## Abstract

This study explores the efficacy of various EEG complexity measures in detecting mind wandering during video-based learning. Employing a modified probe-caught method, we recorded EEG data from participants engaged in viewing educational videos and subsequently focused on the discrimination between mind wandering (MW) and non-MW states. We systematically investigated various EEG complexity metrics, including metrics that reflect a system’s regularity like multiscale permutation entropy (MPE), and metrics that reflect a system’s dimensionality like detrended fluctuation analysis (DFA). We also compare these features to traditional band power (BP) features. Data augmentation methods and feature selection were applied to optimize detection accuracy. Results show BP features excelled (mean area under the receiver operating characteristic curve (AUC) 0.646) in datasets without eye-movement artifacts, while MPE showed similar performance (mean AUC 0.639) without requiring removal of eye-movement artifacts. Combining all kinds of features improved decoding performance to 0.66 mean AUC. Our findings demonstrate the potential of these complexity metrics in EEG analysis for mind wandering detection, highlighting their practical implications in educational contexts.

## Introduction

Mind wandering (MW), defined as the mental activity where thoughts drift away from the primary task or current objectives^1^, is a common psychological phenomenon in daily life^2,3^. Research has extensively documented both the positive and negative impacts of mind wandering on cognitive processes and overall well-being^4,5^. These studies reveal that while mind wandering can foster creativity and problem-solving, it can also detract from focus and productivity.

In the realm of education, video-based learning has emerged as a prominent and oft-used method, offering unparalleled flexibility and global accessibility of educational content. This modality leverages multimedia elements to enhance engagement and comprehension, making it a preferred choice for many^6^. However, despite its advantages, a critical challenge that arises is mind wandering. Studies such as Szpunar et al. highlight the detrimental effects of mind wandering on learning outcomes, emphasizing its significance in educational contexts^7^. A recent study revealed that mind wandering frequency significantly accounted for variance in memory scores, indicating its impact on learning outcomes^8^. Risko et al. explored the prevalence and impact of mind wandering in educational settings, and found that the frequency of mind wandering during video-based learning is higher than classroom learning^9,10^. This phenomenon necessitates a deeper understanding and the development of strategies to mitigate its impact on learning, ensuring that the benefits of video-based learning are fully realized.

The exploration of neural correlates in electroencephalograph (EEG) of mind wandering has been a focus of numerous studies, as highlighted in a recent systematic review by^11^. This review emphasizes the relationship between mind wandering and changes in the amplitude of key sensory and cognitive event-related potential (ERP) components of EEG, such as P1, N1, and P3. In contrast to the relatively consistent patterns observed in ERP measures, spectral markers such as delta, theta, alpha, and beta waves exhibit more variability, though they have been extensively studied in the context of mind wandering.

The above mentioned linear features have been applied to detect mind wandering as well. A study by Dong et al. for instance, used ERP waveform features to detect mind wandering states during an auditory detection task, obtaining above-chance level of decoding performance^12^. This study supports the potential of ERP waveform features in mind wandering detection, although the use of ERP imposes limitations on the types of experimental tasks (and usage scenarios) that can be conducted. To address these limitations, Dhindsa et al. explored the use of band power as features for detecting mind wandering in live lectures^13^, offering an alternative approach that may be more flexible in different learning contexts.

The human brain is a nonlinear system with hierarchical levels that exhibits complex spatiotemporal dynamics across scales during cognitive functions or in disease states. Characterizing the nonlinear evolution of these spatiotemporal patterns is key to understanding brain function^14,15^. This perspective may also extend our understanding of neural bases of mind wandering and the technical question of how to detect it. In the exploration of mind wandering, complexity metrics, a type of nonlinear feature, have emerged as valuable tools for detection and characterization. One such metric is Higuchi’s fractal dimension (HFD), and a study revealed a higher HFD across most EEG electrodes in mind wandering episodes compared to attending visual or auditory perception^16^. Lu and Rodriguez-Larios further included Lempel–Ziv complexity (LZC) and sample entropy (SampEn) in their study^17^, which found a reduction in complexity in mind wandering episodes during a breath focus meditation task. Expanding the scope, Cnudde et al. delved into the relationship between mind wandering and complexity measured by multiscale sample entropy, focusing on Navon’s task^18^. Their findings suggested a primary association between mind wandering and higher complexity at coarser timescales at posterior EEG sites. This led to the conclusion that fluctuations in the complexity of EEG across different timescales may serve as a key characteristic of mind wandering. Further advancements in classifying mind wandering were obtained by Chen et al. using multiscale sample entropy, permutation entropy, dispersion entropy, and a variant called fluctuation-based dispersion entropy^19^. Their classification of EEG data from a sustained attention response task (SART) task yielded a notable 0.71 area under the receiver operating characteristic curve (AUC), highlighting the efficacy of these metrics in distinguishing between mind wandering and non-mind wandering states. Collectively, these studies underscore the potential of complexity metrics in detecting mind wandering and offer some insights into the underlying neural processes.

Despite their widespread application, the selection of complexity metrics in EEG mind wandering research often appears arbitrary. To improve understanding, Lau et al. conducted an extensive review to demystify and systematically categorize various complexity metrics^20^. Their classification divides complexity metrics into two primary types: “predictability” and “regularity.” Predictability metrics, such as HFD and LZC, focus on evaluating the correlations within the temporal evolution of a time series. These can be further subdivided based on whether they assess spatial or temporal dimensionality, providing insights into the evolutionary aspects of a system.

Whereas regularity metrics, typically from the “entropy” family, evaluate the presence of repetitive patterns in time series. Calculation of regularity metrics can be done at single or multiple scales. This classification of methods helps us understanding the types of dynamics these metrics characterize.

Several challenges persist in the development of effective mind wandering detection systems. First, most previous studies on decoding mind wandering from EEG have been conducted in controlled laboratory settings. Given the often limited generalizability of EEG-based models across different studies^21,22^, there is a critical need to design experiments that more accurately reflect real-life conditions for practical daily monitoring applications. Second, the probe-caught method (thought probes) can interrupt the primary task, affecting the quality of data^12^, and the resulting datasets typically have limited sample size and lack diversity. Third, there is a need for more systematic research in using complexity features for detecting mind wandering, particularly their relative merits, their settings, and their interrelationships. For instance, the influence of various scales in multiscale entropy on decoding performance requires further investigation.

In this study, we collected EEG data while participants engaged in video-based learning, and then we detected mind wandering offline using various complexity metrics as features, systematically exploring metrics and their settings. The key contributions of our research are:

- We modify the conventional probe-caught method to increase mind wandering capture rate (the portion of probes which were mind wandering). This was achieved by having an experimenter observe participants’ facial expressions, a potential indicator of mind wandering^23^, and manually triggering extra probes.
- Our systematic examination of various pre-processing pipelines and feature types showed that multiscale permutation entropy (MPE) without eye movement artifact rejection showed comparable decoding performance to band power features on data with eye movement artifact rejection. This simplifies the pre-processing of EEG data, making it easier to implement in a real-world scenario, which underscores the practical potential of complexity features in EEG analysis.
- We combined 7 types of features (6 complexity features plus band power) and applied an information-theoretic feature selection method to obtain a detection accuracy of 0.66 AUC.

The approaches presented here demonstrate feasibility of detecting mind wandering in real-time using EEG in naturalistic environments. Such methods can enable future adaptive educational systems to respond to lapses in attention to improve educational outcomes.

## Methods

### Participants

A total of 28 participants, with average age of 22.8 years (range: 19–35), including 14 males and 14 females, were recruited for this study. None of the participants had a history of neurological disorders, and they all had either normal vision or vision corrected to normal. The experiment received approval from the Ethics Review Committee of the School of Psychology at Beijing Normal University (approval number 20221121118). Each participant provided written informed consent prior to participating in the study. All methods and experimental procedures in this study conformed with the declaration of Helsinki and relevant guidelines and regulations.

### Materials and task

We downloaded three course videos, namely “Computer Networks,” “History of Ancient Egypt,” and “Finance” from a Chinese domestic massive open online course platform (URL: https://www.icourse163.org/). These courses were intentionally designed for beginners and required minimal prior knowledge. Each video had an approximate duration of 90 min. To ensure that participants maintained a suitable level of attention throughout the video, each video was equally divided into five segments. These segments formed the five blocks of the entire experiment. Between each block, participants were given a rest period and proceeded to the next block only when they felt ready to continue. To gauge the participants’ level of interest in each course, they were asked to rate their interest on a 7-point Likert scale, ranging from 1 (not interested at all) to 7 (extremely interested). To ensure that the most interesting video did not overly limit participants’ mind-wandering experiences, we selected the course with the highest interest rating, but excluding those rated at 7, and also confirmed that the participant had not previously taken a similar course. This procedure was done for 24 of the participants, while the first 4 participants watched the video they rated highest.

Videos were presented on an 23.8 inch computer monitor with a resolution of 1920 × 1080 pixels, while the video format was maintained at 480p resolution. For assessing the participants’ learning, we developed a quiz consisting of 10 questions, which included multiple-choice and true/false questions. The distribution of these question types varied across the courses. This quiz was administered both before and after the experiment. Additionally, an 8 min course video, unrelated to the aforementioned courses, was used as practice. Its purpose was to familiarize the participants with the task procedure and ensure they could provide appropriate responses to the probes. On average, participants had interest ratings of 5.21 for the presented courses. The distribution of participants across the courses “Computer Networks,” “History of Ancient Egypt,” and “Finance” was 10:9:9, respectively. In terms of learning outcome, the average pre-test score was 1.38 (out of a total of 10 points), and the average post-test score was 5.71 (for 24 participants; the first 4 participants were not tested).

### Thought probes

In many studies utilizing the probe-caught method, thought probes are typically triggered randomly. Groot et al. computed the reaction time (${RT}_{CV}$) during experiments and used this as a basis for triggering probes, to enhance the likelihood of capturing mind wandering episodes^24^. However, this approach is mainly applicable to tasks similar to the SART. Kaushik et al. implemented a real-life task and relied on a group of experts to determine instances of mind wandering by analyzing videos recorded during the task^22^. An advantage of this approach is that it does not interrupt the task. However, as the authors noted, discerning attention and distraction is challenging since these mental states are private and subjective. Consequently, strong indications of either are needed for accurate annotation, making subjective reports still essential for less obvious instances.

**Figure 1:**
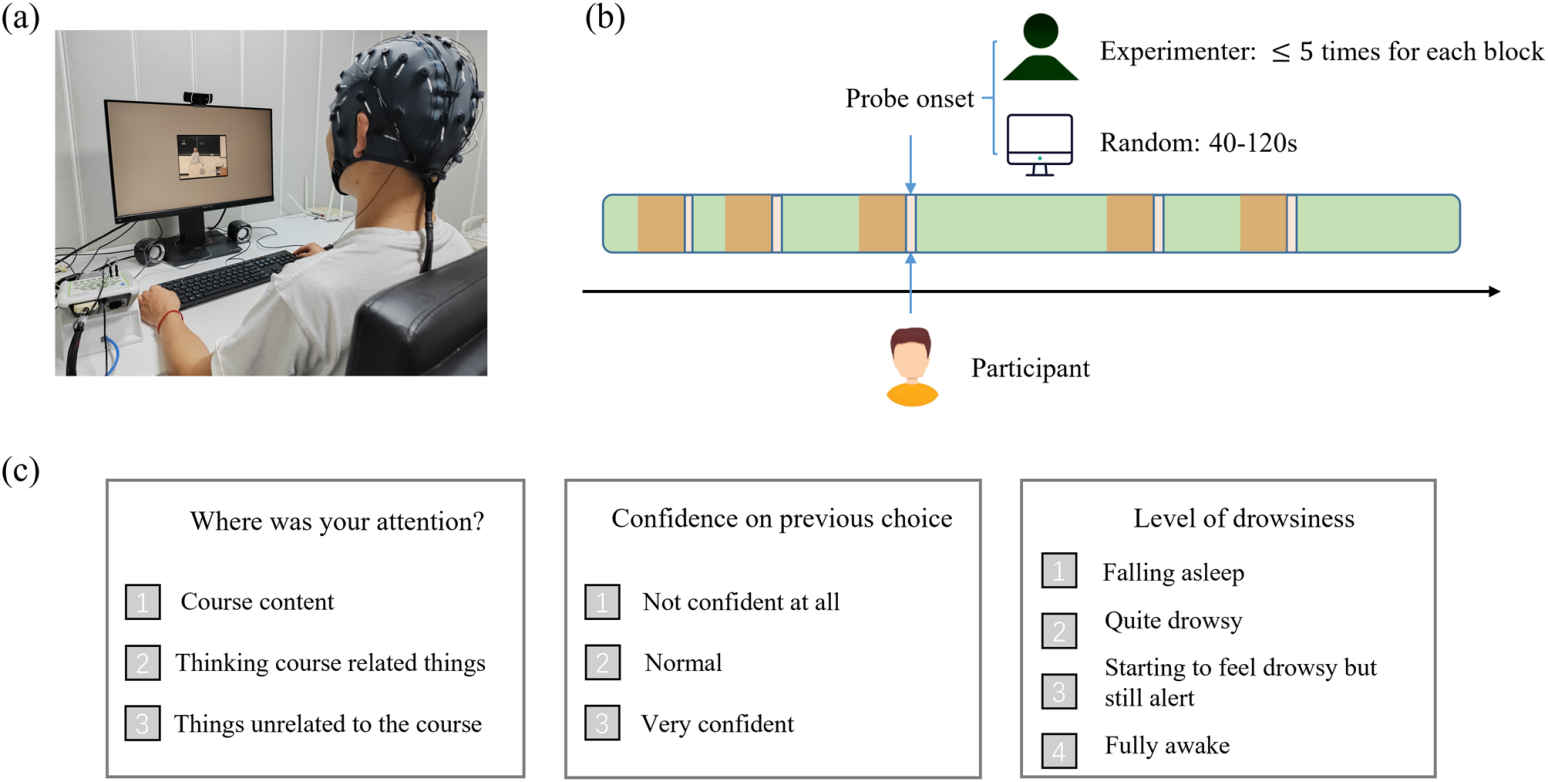
Schematic of the experimental procedure. Participants engaged with educational videos (**a**) and responded to thought probes that appeared on-screen. These probes were triggered at variable intervals ranging from 20 to 120 s, either automatically according to a random schedule or manually by an experimenter monitoring participants’ facial expression (**b**). Upon probe activation, the video paused, and participants were asked to report their state and give a confidence rating (**c**). EEG data collected before the probes (orange shading, duration 17.5 s or 15 s, see Fig. 3.) were labeled as instances of mind wandering (MW) or non-MW based on the participants’ response to the first question (3: MW; 1 or 2: non-MW).

In our experiment, we used two types of probe triggers (Fig. 1). The majority were pre-set to occur randomly within each experimental block, with intervals ranging from 40 to 120 s. The rest were manually triggered by an experimenter who monitored the participants’ facial expressions through a webcam. If clear signs of mind wandering or active engagement were observed, the experimenter initiated a probe using key commands based on their judgment (commands are the same as the participants’ choices for responses). All participants responded to these probes in the same manner, unaware of the triggering method. To avoid overly frequent probes, any that would occur within 20 s of the previous were skipped. Moreover, to keep the probe frequency consistent across blocks and to manage the experimenter’s workload, we capped the number of manually triggered probes at 5 per block. On average, 16.3 probes were triggered in each block (81.3 probes in the whole experiment), and among them, 4.4 were triggered by the experimenter.

Each probe paused the video presentation until the participant’s responses were complete, which was not limited in duration. For each probe, participants responded to the following 3 questions: (i) ‘Where was your attention?’ (3 response options: 1-focusing on the course content, 2-course related things, 3-things unrelated to the course) (ii) ‘Confidence on previous choice?’ (3 options: 1-not confident at all, 2-normal, 3: very confident) (iii) ‘Level of drowsiness?’ (4 options: 1-Falling asleep, 2-quite drowsy, 3-starting to feel drowsy but still alert, 4-fully awake). The mind wandering state concluded from the probe depended on the response to the first question, with 1 (course content) or 2 (related things) designated as non-MW and 3 (unrelated things) designated as MW. Across all participants, 28% of the thought probe reports indicated mind wandering. In our analysis, probes with a confidence rating of 1 were excluded.

### EEG recoding

Electroencephalography (EEG) data were recorded using a 30-electrode Neuroscan Grael system and cap. The electrodes were placed at FP1, FP2, F11, F7, F3, FZ, F4, F8, F12, FT11, FC3, FCZ, FC4, FT12, T7, C3, CZ, C4, T8, CP3, CPZ, CP4, P7, P3, PZ, P4, P8, O1, OZ, and O2 of the International 10–20 EEG system. The cap also included a reference electrode near Cz and a ground electrode on the forehead. Horizontal electrooculography (HEOG) electrodes were also placed to record horizontal eye movements. Vertical eye movements were not monitored due to interference with facial video recording. Two additional electrodes were placed on the left and right mastoids. Impedance of all electrodes was kept below 10 kΩ. The EEG data were acquired continuously with a frequency range from DC to 400 Hz and at a sampling rate of 1024 Hz.

**Figure 2:**
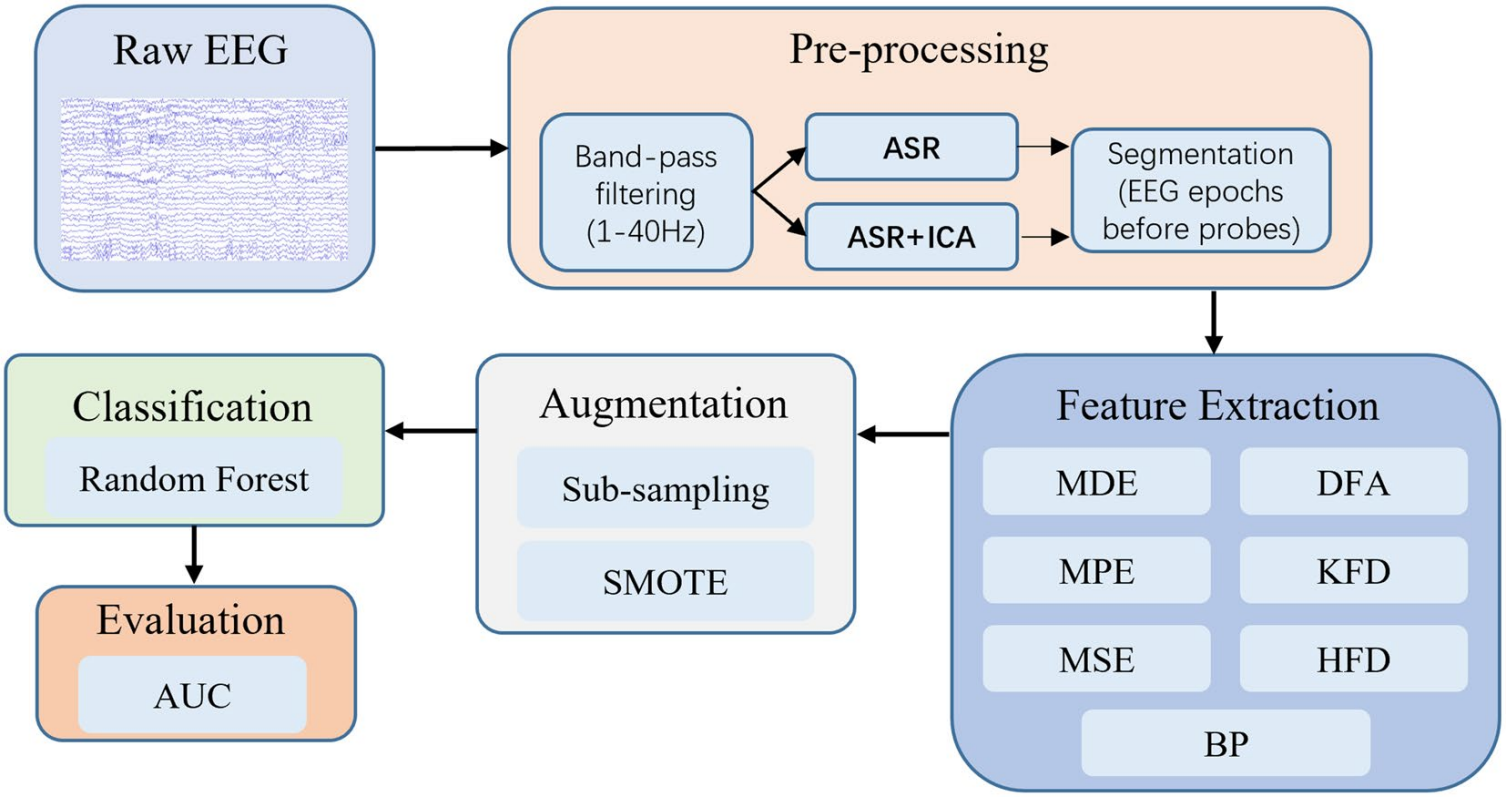
The processing flow of EEG signals. The datasets are differentiated at the pre-processing stage into two types: ASR and ASR + ICA. Epochs preceding probes are extracted from the continuous signal for subsequent feature extraction, with specific details outlined later in the main text. The ASR dataset is processed to address abrupt artifacts, whereas the ASR + ICA dataset additionally utilizes ICA to filter ocular artifacts. Following these initial steps, both datasets undergo an identical processing pipeline for subsequent analyses.

The EEG signal processing in our study involves several distinct steps, as depicted in Fig. 2. The subsequent sections will provide detailed descriptions of each component in this processing sequence.

### EEG signal pre-processing

Custom scripts and the EEGLAB toolbox^25^ in MATLAB were employed for pre-processing. Bandpass filtering between 1 and 40 Hz was applied using the ‘pop_eegfiltnew’ function, and signals were re-referenced to the common average. The filter orders for the high pass filter and low pass filter were automatically estimated by EEGLAB. The signals were then down-sample to 256Hz.

To handle abrupt artifacts, we employed the Artifact Subspace Reconstruction (ASR) method with a cut-off value set to 100. Instead of reconstructing, we chose to delete segments exceeding this threshold. For removing eye-movement components, independent component analysis (ICA) was optionally conducted using EEGLAB’s “runica” function with default settings. The ICLabel plugin was used to label the ICs, and those labeled as ‘Eye’ were removed.

We prepared two versions of the processed datasets for analysis:

- (1) The ASR dataset, which underwent only ASR processing.
- (2) The ASR + ICA dataset, comprising data processed first by ASR and then by ICA.

Then, 10 s windows before probes were extracted for subsequent feature calculation. To increase the number of samples (windows) per condition, multiple (offset) windows per probe were extracted, and they had some degree of overlap (see Sample Augmentation for details).

### Feature extraction

Based on previous studies on mind wandering using complexity measures of EEG and the categories organized by Lau et al.^20^, to describe the predictability of EEG signals, we chose 3 metrics from the “temporal dimensionality” category, and for the regularity of EEG signals, we chose 3 multiscale entropy measures, which capture the complex nature of biological systems better than mono-scale entropy measures. We also included band power features for a comparison to standard methods. Below we give brief introductions to each of the metrics (for details and computation, see supplementary materials).

#### Regularity metrics

Under the regularity category, entropy metrics characterize the repetitive patterns in a signal.

- Sample entropy (SE)^26^: SE works by comparing the number of sequences that match in a time series within a certain tolerance, without including self-matches. A lower SE value indicates more self-similarity or regularity in the data, while a higher value suggests greater complexity or irregularity.
- Permutation entropy (PE)^27^: PE measures the complexity of a time series by examining the patterns or permutations of its values, rather than the original time series. PE is known for its computational efficiency and is often used in real-time monitoring and analysis of complex systems.
- Dispersion entropy (DE)^28^: DE functions by dividing the time series into symbolic sequences that fall into predefined classes (c-classes). DE allows for flexibility in its implementation, with the mapping of time series data to symbolic sequences being possible through various methods, including linear mapping or the normal cumulative distribution function (NCDF), among others. DE is highly regarded for its computational efficiency and its robustness against noise^29^.

The multiscale metrics involve deriving multiple time-series from the original EEG signal, at different time scales, and then calculating metrics for each time scale. This is done by successively averaging neighboring data points within non-overlapping windows^30^. Here, the scales were set to range from 1 to 14 (1 is the original scale without averaging, 2 means averaging 2 neighboring data points, etc.). The derived multi-scale entropies are MDE, MPE, and MSE (for DE, PE, and SE, respectively). Each feature’s dimension was 14 × 30 = 420.

#### Temporal dimensionality metrics

Metrics under the temporal dimensionality group consider the time series as a geometric figure, are fast and efficient, and do not assume stationarity of the signal.

- Higuchi’s fractal dimension (HFD)^31^: HFD calculates the fractal dimension directly from the time series. Fractal dimension (FD) define the minimum number of coordinates needed to locate any point within the phase space and can be interpreted as a measure of the structural complexity of a dynamical system.
- Katz’s fractal dimension (KFD)^32^: KFD also directly calculates the FD from time series data. However, compared to HFD, KFD tends to underestimate the true FD of a system. Despite this, KFD demonstrates robustness against noise and is more effective in distinguishing different brain states, making it useful in certain contexts where discerning these variations is more crucial than precise FD quantification^33^.
- Detrended fluctuation analysis (DFA)^34^: DFA estimates the Hurst exponent, a measure that, like fractal dimension (FD), evaluates complexity but over longer periods. DFA’s distinctive approach focuses on analyzing trends within the data, rather than the overall range of signals. It is effective in analyzing EEG data to understand the temporal correlations in brain activity, which can change during different cognitive states, including mind wandering^35^.

For band power (BP), we extracted delta (1–4Hz), theta (4–8Hz), alpha (8–12Hz), and beta (12–30Hz) bands, as studies mainly found correlations between mind wandering and low-frequency EEG^11^. The EEG was first decomposed by discrete wavelet transform (Daubechies 4 wavelet, 5 levels), then the mean signal power (square of the signal series) was computed^36^. The feature dimension of BP was 4 × 30 = 120, and the dimension of each temporal dimensionality metric was 30.

### Model evaluation

Cross-participant prediction is often used to evaluate the generalizability of classification methods. However, studies report limited generalizability in this context^21,22^. A fMRI study by Groot et al. on mind wandering decoding showed a significant drop in performance when going from within-participant to cross-participant prediction^24^, indicating the substantial impact of individual differences.

**Figure 3:**
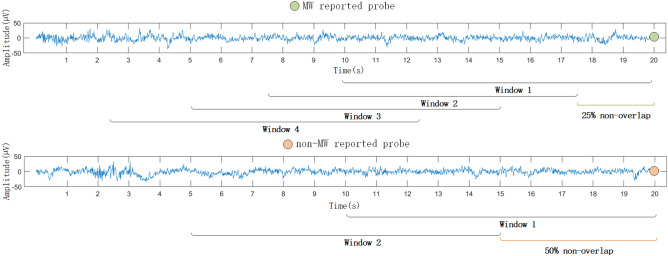
Sample balancing via sub-sampling technique. The cross-validation process is probe based and the windows extracted before each probe may overlap. This creates a denser sampling for probes where mind wandering (MW) is reported, compared to non-MW reported probes, at a ratio of 4:2. The sparser sampling for non-MW probes acted as a sub-sampling strategy, enhancing the sample balance between MW and non-MW instances within the dataset.

Commonly, within-participant k-fold cross-validation is employed, where k-1 folds are used for training and the remaining fold is used for testing. The challenge lies in preventing information leakage while effectively utilizing limited samples. For the probe caught method, per-participant sample sizes are small (due to probe frequency of around 1 each minute). We here use a leave-probes-out cross-validation approach, modifying the conventional within-participant k-fold cross-validation to split training and testing datasets based on probes. This approach allows using several overlapping windows before a probe as EEG data samples to increase sample size without risking information leakage between samples from the same probe. Additionally, this approach helps balance the dataset by allowing us to adjust the number and step size of overlapping windows. The windows can start up to at most 17.5 s before probes, and they end at the time of the probe, at the latest (see Fig. 3).

### Sample augmentation

In the study, participants with a mind wandering sample proportion below 10% were excluded, affecting 3 individuals. Among the remaining 25 participants, and after discarding low confidence responses, the average proportion of mind wandering samples was 27.8%. To balance the training dataset between mind wandering and non-MW, we varied the number and step size of overlapping EEG windows before each probe. Given the rarity of mind wandering reports, we used denser windows (75% overlap, 2.5 s step, 10 s duration, 4 windows) for probes labeled MW and coarser windows (50% overlap, 5 s step, 10 s duration, 2 windows) for probes labeled non-MW (Fig. 3). To address the remaining small sample imbalances, we also applied the synthetic minority over-sampling technique (SMOTE)^37^, generating synthetic instances for the minority class by interpolation (without changing the original instances). For the test set, we consistently used 1 window (10s to 0s) before the probe.

### Feature selection

Small sample sizes paired with high feature dimensionality present difficulties for traditional feature selection techniques. Tsai and Sung^38^ tested various single feature selection methods and ensemble techniques on high dimension, low sample size (HDLSS) data. They found these methods did not improve classification accuracy but significantly reduced the number of features. This reduction is beneficial as it lowers complexity, cost, and risk of overfitting. In our study, prioritizing computational efficiency for practical application, we chose the minimum redundancy and maximum relevance (MRMR) method, which selects features that are highly relevant to the target variable (measured via mutual information) while also being minimally redundant among themselves^39^.

### Classifier and evaluation metric

We treat the detection of mind wandering as a standard two-class classification problem: non-MW versus MW for each EEG data window. Following Chen et al.^19^, we employed a random forest classifier for mind wandering detection (‘RandomForestClassifier’, scikit-learn toolbox^40^, v1.3.2 in Python. The classifier was used with default parameters: ‘n_estimators’, ‘max_depth’, and ‘min_samples_split’ were set to their default values). To ensure robust model performance estimates, we used repeated (2 times) stratified tenfold cross-validation (notice that fold divisions are probe-based). The area under the receiver operating characteristic curve (AUC) was chosen as the evaluation metric, providing a comprehensive view of classification performance across all decision boundaries. AUC combines the entire curve into a single score, where 1 indicates perfect prediction and 0.5 represents chance level. For the number of probes for each participant and the sample size during the cross-validation process, see Table S1 in the supplementary materials.

## Results

### Time-scale for multiscale entropies

**Figure 4:**
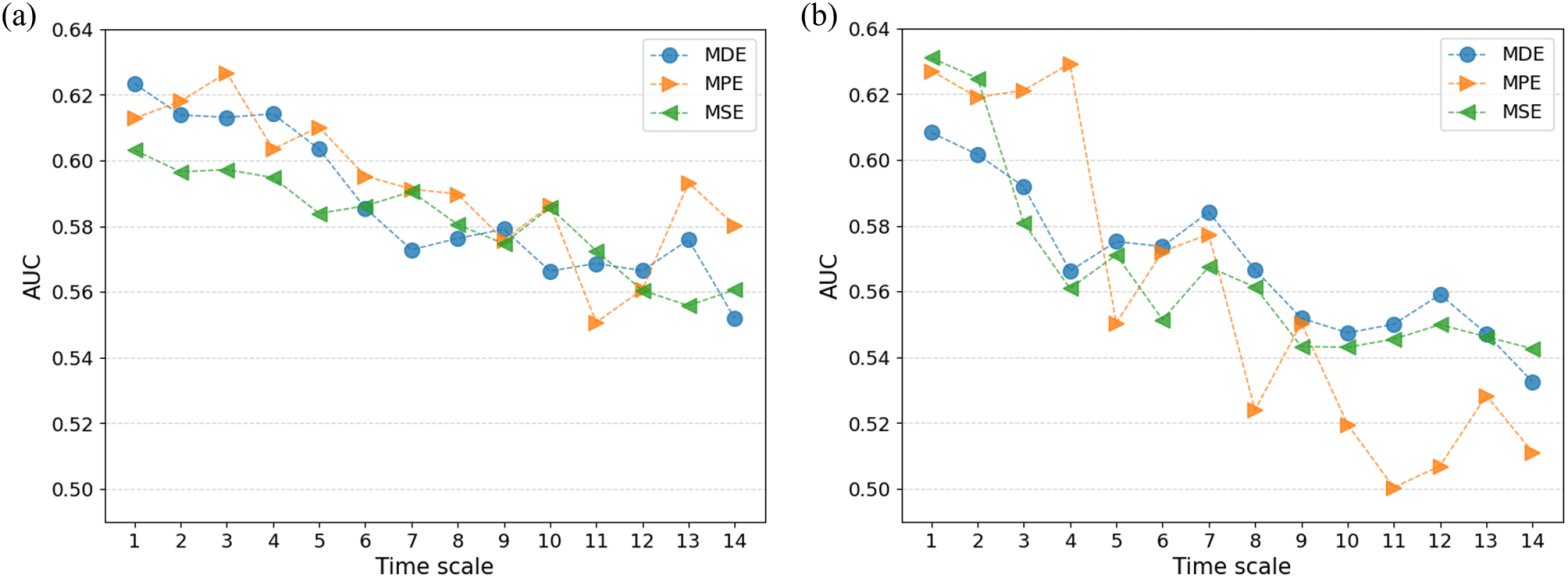
Time scale evaluation for complexity metrics. We evaluated the performance of Multiscale Dispersion Entropy (MDE), Multiscale Permutation Entropy (MPE), and Multiscale Entropy (MSE) across a range of time scales from 1 to 14. The evaluation was conducted separately for (**a**) the ASR-processed dataset and (**b**) the dataset processed with both ASR and ICA.

For multiscale entropy metrics, we systematically compared detection performance for time scales ranging from 1 to 14, classifying using features from a single time scale setting, pooling across all channels. Time scale one corresponds to the scale traditionally used by single-scale methods. We found a trend where detection AUC diminishes as the time scale increases (Fig. 4). Based on this observation, subsequent analyses were confined to time scales from 1 to 10 to use settings with higher efficacy.

### Comparison of features

**Figure 5:**
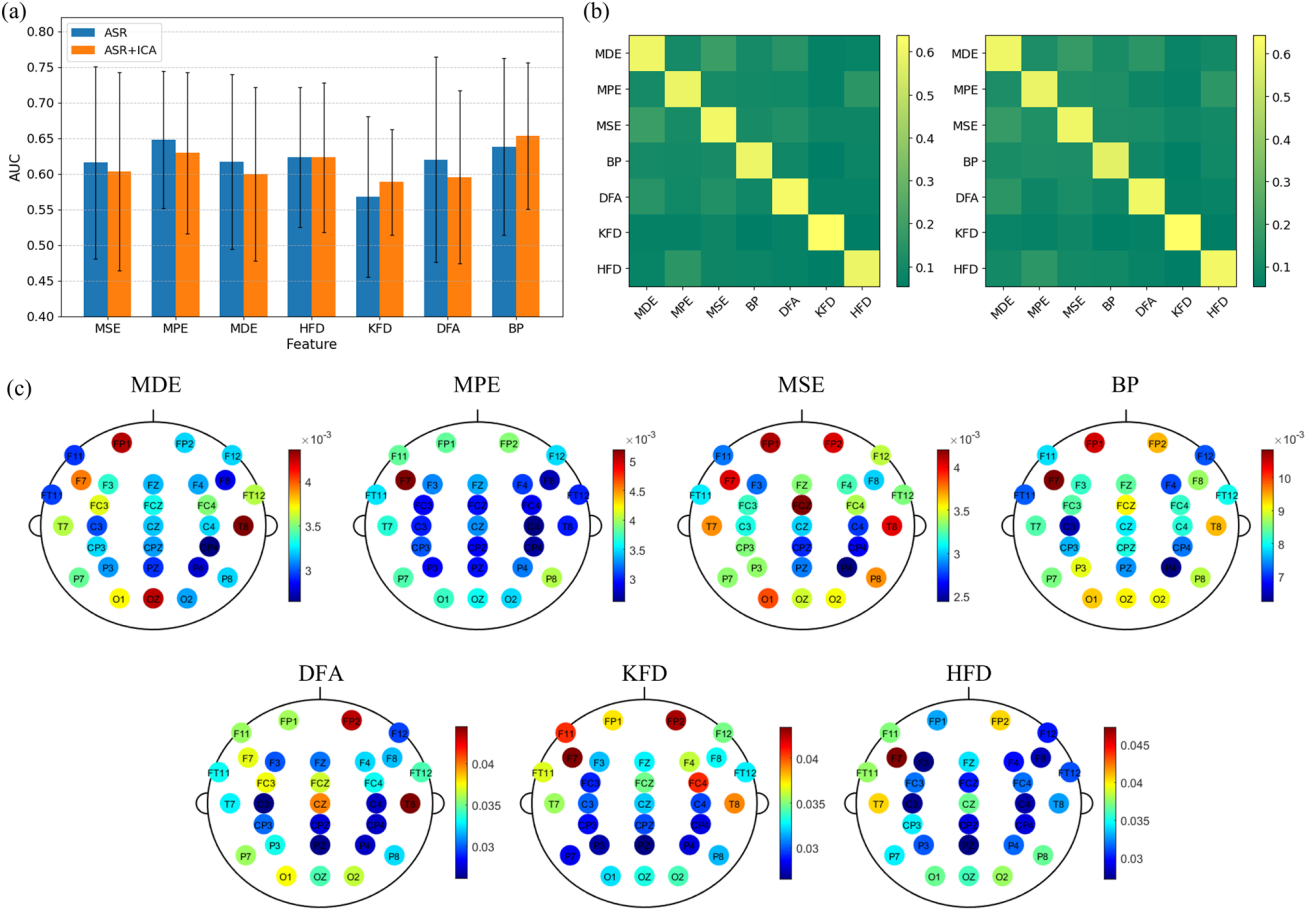
Feature performance and interrelation. (**a**) This graph compares the classification efficacy of models constructed using seven distinct features, with each model utilizing all channels. Error bar represents standard deviation AUC across participants. (**b**) Displayed in a heatmap format, this graph shows the mutual information values among the predictions generated by the models based on the seven different features, illustrating the degree of prediction similarity or uniqueness between them (left: ASR, right: ASR+ICA). (**c**) Feature importance for each channel derived from the fitted random forest models, constructed using seven distinct features on all channels. The values in the colorbars indicate the mean decrease in impurity.

We compared the mind wandering detection performance of the seven features by constructing separate classifiers for each feature type (Fig. 5a). A repeated measures two-way ANOVA, with pre-processing method and feature types as within-participant factors, showed a significant main effect of feature type [F(6,144) = 5.033, p < 0.001]. Among all features, band power exhibited the highest AUC value (0.646), closely followed by MPE with an AUC of 0.639. The pre-processing method did not show a significant main effect [F(1,24) = 0.279, p = 0.602], nor was there a significant interaction between pre-processing method and feature types [F(6,144) = 1.233, p = 0.293]. To assess how similar the predictions were among classifiers using different features types, we computed the mutual information for the prediction labels (Fig. 5b). The mutual information values suggest a low degree of correspondence between the predictions from different feature-based classifiers, indicating a variety of predictions across the feature sets. Additionally, the impurity decrement^41^ (a metric in random forest parameter fitting) was extracted from each fitted random forest model, and the mean values were computed for each channel to represent their respective importance. The results are presented in Fig. 5c. Notably, the spatial distribution of channel importance exhibits variations across feature types, with consistent higher importance observed in the frontal polar area (FP1 and FP2) and occipital area (O1, Oz, and O2).

### Feature selection

We next explored feature selection and the number of features which gives good performance. We tested various feature set sizes and employed minimum redundancy maximum relevance (MRMR), a mutual-information based method^39^, to automatically select features. Figure 5b suggests that predictions generated by classifiers based on different feature types are largely distinct, suggesting that integrating these features could be beneficial. Thus, we examined the detection accuracy when pooling all features of different types together. Since there are no significance difference between pre-processing pipelines, we choose the ASR dataset for further analysis.

**Figure 6:**
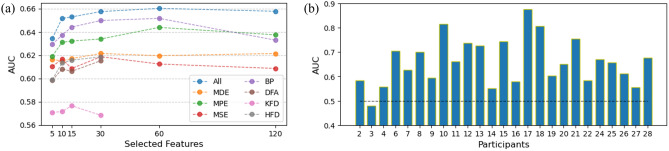
Feature number and performance across participants. (**a**) For each feature type, we tested different numbers of features selected by MRMR. “All” is where all types of features are pooled together for selection. Notice that DFA, KFD, and HFD have only 30 features. (**b**) Individual AUC scores using 60 features selected from all types shows substantial individual variability. Dashed line is chance level.

The comparison of mean AUC values for classifiers based on individual features or pooled together (referred to as All) is illustrated in Fig. 6a. Notably, among these, the AUC of the All and BP classifiers stands out as relatively higher. A repeated measures two-way ANOVA was conducted, with feature type (All and BP) and selected features considered as within-participant factors. The analysis revealed a significant main effect of selected features, F(5, 120) = 4.081, p = 0.002. However, the main effect of feature type did not reach significance, F(1, 24) = 0.695, p = 0.413. Additionally, the interaction effect was not statistically significant (F(5, 120) = 0.817, p = 0.54).

When examining the AUC per participant, we employed a configuration involving 60 features selected from All feature types, the detection performance had substantial individual variability (Fig. 6b), with approximately one-third of the participants having an AUC greater than 0.7.

### Channel comparison

**Figure 7:**
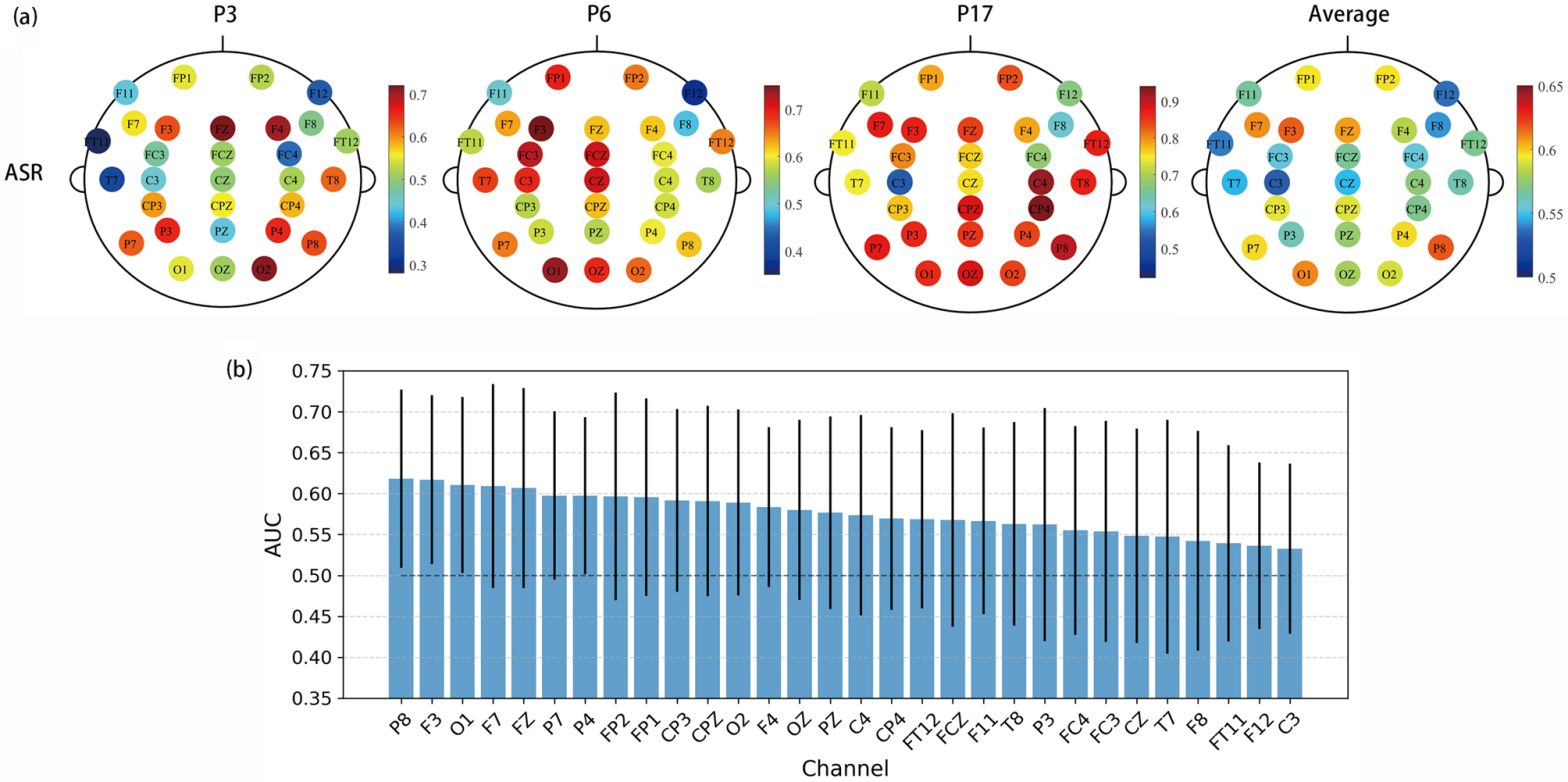
(**a**) AUC scores among all channels (ASR dataset) for three example individuals (participant 3(P3), 6(P6) and 17(P17)) and participant average. (**b**) Channel-wise decoding performance sorted by AUC.

To determine if certain brain regions (channels) are particularly effective for detecting MW, we performed classification based on individual channels. The features included all 7 kinds of features, and no feature selection was performed. Figure 7a displays the single-channel classification AUC, for 3 example individuals, alongside the average performance across all 25 participants. We chose these 3 participants as they are representative of participants with low (P3), medium (P6), and high (P17) AUC. Figure 7b shows mean ± one standard deviation of AUC for each channel (averaged across participants), sorted by AUC. The results in Fig. 7a and Fig. 7b suggest that there is considerable variation among individuals regarding the effectiveness of single channels for MW detection. The variation includes both the number of channels yielding higher performance and the spatial distribution of these channels across the scalp.

## Discussion

In our study aimed at detecting mind wandering during video-based learning, we designed an experimental task where participants watched course videos while we recorded their EEG signals. We designed the experiment and data analysis pipeline to increase the number of data samples with mind wandering, creating a more balanced dataset, which helps classifier training. We systematically compared complexity-based features, settings for these features (time scales for multiscale entropies, number of features), and brain regions (EEG channels). Our findings suggest that complexity metrics, particularly multiscale permutation entropy (MPE), require less pre-processing and are equally effective compared to band power features that require more pre-processing (ICA to remove eye-movement artifacts). Pooling features and using feature selection by mutual information, we obtained an average AUC of 0.66, demonstrating the potential of these methods for detecting mind wandering in educational contexts. However, our analysis of per-participant and per-channel detection performance showed a large influence from individual variability.

We observed a relatively low mind wandering rate, averaging 28% of probes (a comparison of mind wandering rates for the first and second halves of each block is illustrated in Fig. S1 in the supplementary materials). This sample imbalance presents a significant challenge for training classifiers, since the majority class (non-mind wandering) can dominate the classifier training process, leading to a bias in prediction towards this majority class^42^. When we compare our findings with other studies that employed EEG and thought probes to measure mind wandering rates, notable variations emerge based on the nature of the tasks involved. For instance, in Navon’s task, participants reported experiencing mind wandering 45% of the time^18^, while in an auditory target detection task, the rate was reported at 55%^12^. In the sustained attention to response task (SART), the rate was 43%^24^. During a live lecture, the mind wandering rate was 34.7%^13^. Interestingly, in the Tibetan Monastic debate task, only 26 out of 46 participants reported a singular type of mind wandering experience^22^. Overall, the reported rates seem to be related to the realism of the experimental task, and our results are more similar to those tasks with more realistic experimental settings, which tend to elicit lower mind wandering rates. This may be due to the relatively less monotonous nature of realistic tasks, wherein participants have higher motivation to participate.

In our experimental design, we aimed to improve the efficiency of sampling neural data during mind wandering while minimizing disturbances to participants. To achieve this, we triggered probes manually for a portion of the probes. Compared to other modifications for the probe-based method^24^, the efficacy of this modification can be evaluated, since manually triggered probes have accompanying judgements (made by the experimenter) of the mind wandering state, and these have corresponding true answers (i.e., state reported by the participant). The behavioral results indicate that this approach yielded an accuracy rate of $0.53\pm 0.19$ (baseline rate was 0.46, obtained by shuffling). This number can be thought of as the ability of the experimenter to judge mind wandering state from facial expression. Our findings demonstrate that the human accuracy for detecting mind wandering exceeded random chance, although there was considerable variability among participants. In this study, the manual probe triggering method served as a complementary approach to random probes. The manual triggers were given by a single experimenter; this aspect of our design could be further refined in future studies to enhance effectiveness.

The accuracy in most mind wandering detection studies typically exceeds random chance by a small margin, and they commonly use linear features^12,13,24^. However, Chen et al. had substantially better results, attaining a 0.71 AUC for cross-participant prediction through entropy-based features^19^. In our study, we explored several complexity-based features and found that MPE was most effective, particularly when used on data that were not processed to remove eye-movement artifacts. However, our overall detection AUC was slightly lower than their results. We believe this marginal difference in performance can be attributed primarily to the more ecologically valid experimental setting of our study, which tends to amplify the impact of individual differences (e.g., the different video courses). We note that mind wandering episodes tend to occur more frequently in the second half of blocks, with higher reported rates in the later blocks (Fig. S1). This observed trend may be influenced by factors such as fatigue and a potential learning effect (where increased knowledge leads to reduced curiosity). Since time on task can alter the properties of the EEG signal, we acknowledge the potential impact for decoding performance, but since the temporal imbalance was not very large, we believe the effect on our results is minor.

Ocular artifacts in EEG has been a longstanding issue within the EEG community^43^, particularly in various neuroscience research. However, for mind wandering detection, the conventional view on the detrimental impact of ocular artifacts is shifting. Several studies have identified correlations between mind wandering and fixation^44^ and blinks^45,46^. Consequently, including ocular ‘artifacts’ in the EEG signal may enhance decoding performance^19^. Moreover, due to reduced need for preprocessing steps, this approach may be more practical for real-world applications. Our results indicate that multiscale permutation entropy (among the tested metrics) stands out as a particularly effective way to extract informative features in signals from both brain and ocular sources.

Our findings revealed that classifiers using different types of features yielded relatively distinct predictions, as indicated by low mutual information values between them (Fig. 5b). However, combining all types of features resulted in only a slight improvement. This outcome suggests that the information captured by EEG might be insufficient or limited in scope (especially in a realistic setting). Notably, studies employing other modalities, such as video and eye-tracking, have also been applied to detect mind wandering^23,47,48^. Integrating multiple modalities could potentially lead to higher detection accuracy and is a promising direction for future research. Moreover, the current study employed handcrafted features that necessitate human expertise, such as determining the time scale for regularity metrics. This manual approach may potentially overlook informative features. Leveraging automated feature extraction through deep learning has the potential to improve decoding performance^49,50^. Additionally, interpretable convolutional neural networks can offer valuable scientific insights into the neural basis of mind wandering^51–53^.

### Supplementary Information

Supplementary Information.

## Data Availability

The measurement data supporting the conclusions of this article will be made available by the corresponding author, without undue reservation.
