# Supplementary material for: EEG complexity measures for detecting mind wandering during video-based learning
Source: Sci Rep. 2024 Apr 8;14:8209. doi: 10.1038/s41598-024-58889-9 (PMC11001605; doi:10.1038/s41598-024-58889-9)
Supplement: Supplementary file 1 — Supplementary Information. [file 41598_2024_58889_MOESM1_ESM.docx]

# Supplementary Materials

Here we detail the methods for calculating sample entropy (SE), permutation entropy (PE), dispersion entropy (DE), Higuchi's fractal dimension (HFD), Katz's fractal dimension (KFD), and detrended fluctuation analysis (DFA). Additionally, we provide information on the key parameters used and the software toolboxes employed in these calculations.

For SE, PE, and DE, calculations were performed using the 'EntropyHub' Python package, available on GitHub at https://github.com/MattWillFlood/EntropyHub. Meanwhile, the calculations for HFD, KFD, and DFA were conducted using the 'Antropy' Python package, accessible at <https://github.com/raphaelvallat/antropy>.

**Sample entropy** ^1^

$$Sample entropy(m,r,N)=-ln (\frac{A(m+1,r)}{A(m,r)})$$

$N$: the length of the time series;

$m$: template length (embedding dimension). This is set to 2 in the current study;

$r$: the tolerance or filtering parameter, indicating the maximum difference allowed between two similar sequences. This is set to 0.2 times the standard deviation of the time series in the current study.

$A(m,r)$ is the total number of template vectors of length $m$ that have similar vectors (within the tolerance $r$) in the time series.

**Permutation entropy** ^2^

PE quantifies the complexity of a time series by analyzing the order relations between its values. It does this by first transforming the time series into a sequence of symbolic permutations, then it calculates the Shannon entropy of these permutations.

$$Permutation entropy\left( m \right)=-\sum_{i=1}^{m!} p_{i}{log}_{2}p_{i}$$

$m$: template length. This is set to 3 in the current study;

$p_{i}$: the number of times the $i$th permutation was found in the time series divided by the total length of the sequence.

**Dispersion entropy** ^3^

The calculation of dispersion entropy can be separated into 4 steps:

1. Discretize the continuous time series into $c$ classes. For example, using the normal cumulative distribution function (NCDF), map the values from input signal into range 0 to 1, and then monotonically assign each mapped value to an integer from 1 to $c$.
2. Construct embedding vectors (time series), each vector is:

$$z_{i}^{m,c}=\left\{ z_{i}^{c},z_{i+d}^{c},\ldots,z_{i+(m-1)d}^{c} \right\}$$

$$i=1,2,\ldots,N-(m-i)d$$

where $m$ is embedding dimension and $d$ is the time delay.

Then map the vector $z_{i}^{m,c}$ to a dispersion pattern $\pi_{v_{0}v_{1}\ldots v_{m-1}}$, where $z_{i}^{c}=v_{0},\ldots,z_{i+(m-1)d}^{c}=v_{m-1}$. There are $c^{m}$ possible dispersion patterns to the vector $z_{i}^{m,c}$.

1. Calculate relative frequency for each dispersion pattern:

$$p\left( \pi_{v_{0}v_{1}\ldots v_{m-1}} \right)=\frac{Number\{i|i\leq N-\left( m-1 \right)d, z_{i}^{m,c} has type \pi_{v_{0}v_{1}\ldots v_{m-1}}\}}{N-(m-1)d}$$

2. $DispEn$ of a time series $\boldsymbol{x}$ is calculated as:

$$DispEn\left( \boldsymbol{x},m,c,d \right)=-\sum_{\pi=1}^{c^{m}} p\left( \pi_{v_{0}v_{1}\ldots v_{m-1}} \right) ln(p(\pi_{v_{0}v_{1}\ldots v_{m-1}}))$$

We used a linear data-to-symbol sequence transform; $m$ and $c$ were set to 3 in the current study.

**Higuchi's fractal dimension** ^4^

Given $N$ points-long signal $X$, and a parameter called maximum delay $k_{max}\geq2$,

for each $k\in\{1,2,..,k_{max}\}$ and $m\in\{1,2,..,k\}$, the length of $L_{m}\left( k \right)$ is defined as:

$$L_{m}\left( k \right)=\frac{N-1}{\left\lfloor\frac{N-m}{k} \right\rfloor k^{2}}\sum_{i=1}^{\left\lfloor\frac{N-m}{k} \right\rfloor} \left| X_{N}\left( m+ik \right)-X_{N}\left( m+(i-1)k \right) \right|$$

The length $L(k)$ is defined as:

$$L\left( k \right)=\frac{1}{k}\sum_{m=1}^{k} L_{m}\left( k \right)$$

The HFD is derived from the slope of the linear fit to $\left\{ \left( log\frac{1}{k},logL(k) \right) \right\}$. ^5^

In the current study we set the $k_{max}$ to 10.

**Katz’s fractal dimension** ^6^

The key equation in Katz's method is^7^:

$$D=\frac{{log}_{10}\left( N \right)}{{log}_{10}\left( \frac{d}{L} \right)+{log}_{10}\left( N \right)}$$

$D$: Katz's fractal dimension;

$N$: the total number of steps in a grid covering the shape (the graphical representation or trajectory of the time series data when plotted in space);

$d$: the diameter of the shape, or the longest distance between any two points on the shape;

$L$: the total length of the shape's outline.

In simpler terms, Katz's fractal dimension ($D$) is calculated by comparing the length of the shape's outline ($L$) to the size of the shape ($d$), and how many steps ($N$) are needed to cover the shape. The logarithms (${log}_{10}$) help handle very large or small numbers more easily.

**Detrended fluctuation analysis** ^8^

The computation of DFA generally follows these steps: ^9^:

1. Integrating the time series after subtracting the mean.
2. Dividing the integrated time series into segments of equal length.
3. In each segment, fitting a polynomial trend and subtracting it from the integrated time series (detrending).
4. Calculating the root mean square fluctuation of this detrended integrated time series.
5. Repeating these steps for various segment lengths to find the relationship between the average fluctuation and segment length.

We used the default parameters of the 'Antropy' pakage.

# References

1. Richman, J. S., Lake, D. E. & Moorman, J. R. Sample Entropy. in *Methods in Enzymology* vol. 384 172–184 (Academic Press, 2004).

2. Bandt, C. & Pompe, B. Permutation Entropy: A Natural Complexity Measure for Time Series. *Phys. Rev. Lett.* **88**, 174102 (2002).

3. Rostaghi, M. & Azami, H. Dispersion Entropy: A Measure for Time-Series Analysis. *IEEE Signal Processing Letters* **23**, 610–614 (2016).

4. Higuchi, T. Approach to an irregular time series on the basis of the fractal theory. *Physica D: Nonlinear Phenomena* **31**, 277–283 (1988).

5. Kawe, T. N. J., Shadli, S. M. & McNaughton, N. Higuchi’s fractal dimension, but not frontal or posterior alpha asymmetry, predicts PID-5 anxiousness more than depressivity. *Sci Rep* **9**, 19666 (2019).

6. Esteller, R., Vachtsevanos, G., Echauz, J. & Litt, B. A comparison of waveform fractal dimension algorithms. *IEEE Transactions on Circuits and Systems I: Fundamental Theory and Applications* **48**, 177–183 (2001).

7. Wijayanto, I., Rizal, A. & Humairani, A. Seizure detection based on EEG signals using katz fractal and SVM classifiers. in *2019 5th International Conference on Science in Information Technology (ICSITech)* 78–82 (IEEE, 2019).

8. Peng, C.-K. *et al.* Mosaic organization of DNA nucleotides. *Phys. Rev. E* **49**, 1685–1689 (1994).

9. Hirekhan, S. R., Manthalkar, R. & Phutke, S. The detrended fluctuation analysis of EEG signals: a meditation-based study. in *Computing, Communication and Signal Processing: Proceedings of ICCASP 2018* 771–780 (Springer, 2019).

**Table S1.** The distribution of classes (MW, non-MW) for probes (in the experiment) and for samples (in the data analysis, the sample size during ‘leave probes out’ cross validation). Please note that the sample sizes in the 'samples' columns apply only to the training set (as the subsampling and SMOTE method were only applied to the training set) during the 10-fold cross-validation process. Non-MW sample size was the same as the MW sample size.

| **Participant** | **non-MW probes** | **MW probes** | **Total probes** | **MW samples** | **Total samples** |
| --- | --- | --- | --- | --- | --- |
| 2 | 51 | 26 | 77 | 77 | 154 |
| 3 | 54 | 18 | 72 | 87 | 174 |
| 4 | 29 | 27 | 56 | 91 | 182 |
| 6 | 54 | 30 | 84 | 104 | 208 |
| 7 | 56 | 21 | 77 | 98 | 196 |
| 8 | 62 | 13 | 75 | 104 | 208 |
| 9 | 64 | 17 | 81 | 116 | 232 |
| 10 | 34 | 47 | 81 | 168 | 336 |
| 11 | 47 | 33 | 80 | 104 | 208 |
| 12 | 71 | 11 | 82 | 125 | 250 |
| 13 | 51 | 25 | 76 | 94 | 188 |
| 14 | 71 | 19 | 90 | 116 | 232 |
| 15 | 55 | 31 | 86 | 108 | 216 |
| 16 | 67 | 12 | 79 | 88 | 176 |
| 17 | 58 | 10 | 68 | 102 | 204 |
| 18 | 52 | 19 | 71 | 92 | 184 |
| 19 | 62 | 14 | 76 | 114 | 228 |
| 20 | 67 | 9 | 76 | 118 | 236 |
| 21 | 63 | 16 | 79 | 93 | 186 |
| 22 | 38 | 39 | 77 | 140 | 280 |
| 24 | 66 | 10 | 76 | 93 | 186 |
| 25 | 45 | 26 | 71 | 79 | 158 |
| 26 | 47 | 30 | 77 | 100 | 200 |
| 27 | 56 | 20 | 76 | 97 | 194 |
| 28 | 54 | 18 | 72 | 75 | 150 |
| **Average** | **55** | **22** | **77** | **103** | **206** |


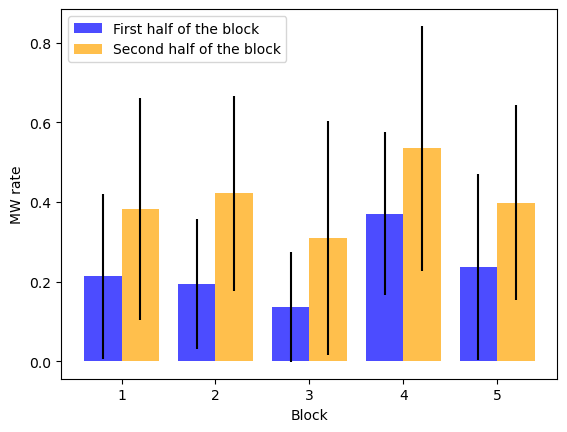


**Figure S1.** Mind-wandering (MW) rates for the first and second halves of experiment blocks. Error bars indicate the standard deviation of MW rates across participants.
